# A novel strategy for forensic age prediction by DNA methylation and support vector regression model

**DOI:** 10.1038/srep17788

**Published:** 2015-12-04

**Authors:** Cheng Xu, Hongzhu Qu, Guangyu Wang, Bingbing Xie, Yi Shi, Yaran Yang, Zhao Zhao, Lan Hu, Xiangdong Fang, Jiangwei Yan, Lei Feng

**Affiliations:** 1 Institute of Forensic Science, Key Laboratory of Forensic Genetics, Ministry of Public Security, Beijing, China; 2 Key Laboratory of Genome Sciences and Information, Beijing Institute of Genomics, Chinese Academy of Sciences, Beijing, China; 3 People’s Public Security University of China, Beijing, China

## Abstract

High deviations resulting from prediction model, gender and population difference have limited age estimation application of DNA methylation markers. Here we identified 2,957 novel age-associated DNA methylation sites (*P* < 0.01 and R^2^ > 0.5) in blood of eight pairs of Chinese Han female monozygotic twins. Among them, nine novel sites (false discovery rate < 0.01), along with three other reported sites, were further validated in 49 unrelated female volunteers with ages of 20–80 years by Sequenom Massarray. A total of 95 CpGs were covered in the PCR products and 11 of them were built the age prediction models. After comparing four different models including, multivariate linear regression, multivariate nonlinear regression, back propagation neural network and support vector regression, SVR was identified as the most robust model with the least mean absolute deviation from real chronological age (2.8 years) and an average accuracy of 4.7 years predicted by only six loci from the 11 loci, as well as an less cross-validated error compared with linear regression model. Our novel strategy provides an accurate measurement that is highly useful in estimating the individual age in forensic practice as well as in tracking the aging process in other related applications.

Aging is a natural and un-preventable process in human life, and its rates are influenced by heredity, environment, lifestyle, and disease. In forensic science, accurate estimation of the age of a victim or suspect can facilitate the investigator to narrow a search and aid in solving a crime. The traditional way of age estimation in forensic science is mainly through morphology of skeletal remains^1^. This is useful only in cases where the skeletal remains are available and relatively intact, and moreover, often requires experienced experts. In most cases, the perpetrator has fled after committing a crime, only fragmentary remains are found, such as some biological evidence including blood, saliva, or tissue. Forensic DNA analyses have allowed scientists to obtain unique genetic profiles of individuals from DNAs extracted from these specimens, which then can be used to identify the suspect. Yet, when the suspect cannot be identified, predictions of physical characteristics such as gender and age become very important in the investigation^2^. Molecular biomarkers may be applied to resolve this problem. Molecular biomarkers such as telomere length, amino acid racemization, mitochondrial DNA mutation, accumulation of advanced glycation end-products (AGEs)^3^, RNA biomarkers^4^, and somatic gene rearrangements^5^ have been found to be correlated with age^6^,^7^,^8^,^9^. However, all of these biomarkers have relatively low precision and practical limitations^9^.

The epigenetic landscape provides new perspectives for using as biomarkers with the development of experimental technologies mediated by high-throughput sequencing and omics data mining^10^,^11^. One of these epigenetic modifications, DNA methylation, has been shown to closely correlate with age, that is, the global level of methylated genomic DNA decreases as a person ages^12^,^13^. It is also reported, however, that at some specific CpG sites, 5-methyldeoxycytidine (5mC) modification increases with age, whereas at other CpG sites 5mC level decreases with age^14^,^15^. Several studies have built age prediction models using DNA methylation with an average error at 2.9–5.2 years^16^,^17^,^18^. A multivariate linear model was recently established to predict age based on 102 CpG sites with a mean absolute deviation of only 3.34 years, based on DNA methylation profiles of 575 Caucasians individuals^18^. However, the linear regression model that these studies mainly used is too simple to explain the complicated relationship between DNA methylation and age.

To this end, we aimed to develop a robust new model based on a limited number of sampling set of loci without cost of loss of accuracy in age estimation for a given population. Genome-wide DNA methylation profiles from eight pairs of Chinese Han female monozygotic twins were analyzed to identify age-associated CpG sites. After validating these profiles in 50 unrelated Chinese Han female volunteers aging between 20 and 80, 11 CpG loci were selected to build the age prediction model by using different models including multivariate linear regression, multivariate nonlinear regression, back propagation (BP) neural network, and support vector recognition (SVR). Based on comparative analyses integrating different loci and models, we established an age prediction strategy based on six CpG loci and SVR model with an average predictive accuracy at 4.7 years. This strategy has a potential value in both forensic practice and other related studies not only for Chinese population, but for other populations as well.

## Results

### Identification of age-associated CpG sites

We first analyzed methylome-wide profiles from whole blood samples of eight pairs of Chinese Han female monozygotic twins aged between 21 and 32 years (Supplementary Table 1) using Illumina HumanMethylation450 BeadChip that can measure the methylation status of 485,577 CpG loci covering 99% of RefSeq genes at single nucleotide resolution. For each CpG site on the chip, we calculated the beta value, which refers to the fraction of methylated cytosines at that location. A beta value of 1 represents a site that is completely methylated on both alleles in all cells, whereas a beta value of 0 means completely unmethylated at that site.

**Figure 1:**
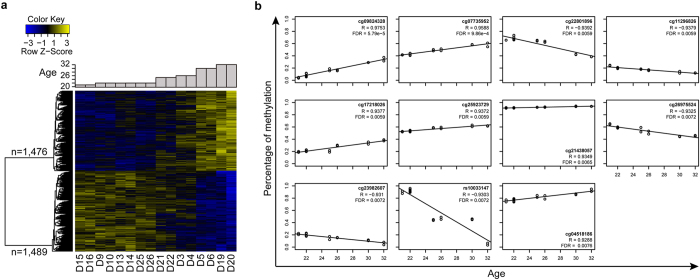
Age-associated DNA methylation sites in human blood of twins, as detected by Illumina Human Methlation540 BeadChip. (**a**) A heatmap of 2,965 age-associated methylation markers selected from eight pairs of female twins under the criteria of *P* < 0.01 and R^2^ > 0.5. The age-associated markers clustered into positive (n = 1,476, the top block) and negative (n = 1,489, the bottom block) correlated markers. The methylation values of each probe were normalized among 16 female samples, which were indicated in blue (low) to yellow (high). The ages for 16 females are shown at the top of the heatmap. (**b**) Scatter plots of methylation value versus age for 11 strongly age-associated DNA methylation sites under more stringent criteria of FDR < 0.01. Out of these, six CpG sites were positive associated with age and five CpG sites were negatively associated with age.

To identify age-associated methylation sites, we calculated the Pearson correlation coefficient (R) and performed a linear regression between age and methylation (beta scores) for each CpG site. Statistical significance of the regression coefficient was determined by *t*-test. With the criteria of *P* < 0.01 and R^2^ > 0.5, we obtained 2,965 age-associated probes (Supplementary Table 2), corresponding to 1,783 genes. Among them, 1,476 CpG sites were positively associated with age and 1,489 markers were negatively associated with age (Fig. 1a). Of these sites, 2,050 (69.1%) fell within CpG islands, which were enriched in these sites relative to the total array of probes (64.7% in CpG islands, *P* = 7.391 × 10^−10^, Fisher’s exact test). Functional analyses showed that the 1,783 corresponding genes were associated with iron binding, hormone receptor binding, voltage-gated ion channel activity, and histone or protein methyltransferase activity compared with genes corresponding with the rest of total loci (Supplementary Table 3). Due to the diversity of populations, platforms, prediction methods, and selection criteria, a total of 88 sites of these 2,965 sites were reported by previous ten similar studies^14^,^16^,^17^,^18^,^19^,^20^,^21^,^22^,^23^,^24^(Supplementary Table 4).

**Figure 2:**
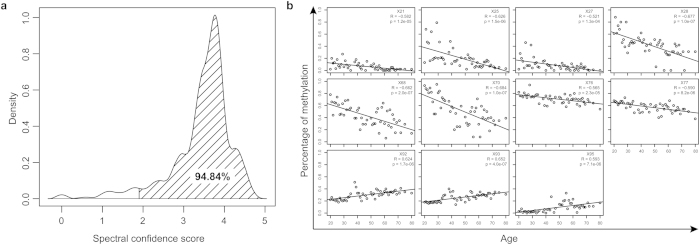
Validation of age-associated methylation sites by using Sequenom MassARRAY in 50 healthy females. (**a**) The reliability of the Sequenom MassARRAY output data. The confidence of methylation values for each product of primer per sample was assigned to a value referring to low (0) to high confidence (5). This value > 1.9 showed that the methylation level can be accepted. Our data from Sequenom MassARRAY is of high quality since 95% values were accepted. (**b**) Scatter plots of the methylation level as a function of age for 11 CpG sites that were selected from the Sequenom MassARRAY result at |R| < 0.5. The ID of CpG sites and their R values are shown in the right top corner of each sub-figure.

For further validation of these age-associated markers in additional females, we selected a more stringent significance level at which the false discovery rate (FDR) is less than 0.01 to reduce the number of loci and only obtained 11 methylated sites highly associated with age including six positive and five negative correlated (Fig. 1b). None of these 11 sites were reported by previous studies. Among them, rs10033147 is a SNP (A/G single base mutation), which means that this CpG site only exists in a part of the population with the allele that has a G base in this position. The primers to amplify the cg11296826 site could not be designed to avoid the presence of CG dinucleotides (Supplementary Fig. 1). DNA within 600 bp of the cg11296826 site (the PCR products for Sequenom should be within a range of 200–600 bp) is in a CpG island. After bisulfate conversion, unmethylated cytosines changed to uracil while methylated cytosines remained cytosines. According to the principle of complementary base pairing, primers containing CG dinucleotides only partially match the DNA template and lead to inaccurate DNA amplification. Therefore, we detected the remaining nine selected highly age-associated markers as well as three markers from the recently published study^18^ (cg02228185, cg25809905, and cg17861230) in blood samples from 50 unrelated Han Chinese females aged between 20 and 80 years, using the Sequenom MassARRAY Platform (for information on primers see Supplementary Table 5). A total of 95 CpG sites were covered in the amplified PCR products by using 12 pairs of primers (Supplementary Table 6). The correlation between the methylation of DNA standards (known 0, 10, 20, 30, 40, 50, 60, 70, 80, 90, and 100% methylated) and their measured methylation levels was linear (R^2^ = 0.8836). However, they did not disperse along the diagonal especially for low-to-medium methylated standards. Therefore, we corrected our data by the linear regression: y = 2.285x −1.2176, where x was the methylation levels measured by Sequenom and y was the corrected data. Meanwhile, 95% of the confidence scores of 95 CpG sites using Sequenom were greater than the cut-off (Fig. 2a).

During aging process, methylation level of some CpG sites has been shown to be either specifically increased or decreased based on their locations, which provides us a plausible hypothesis that a small subset of age-associated methylation sites might be sufficient for age prediction. By analyzing the Sequenom detection dataset we have generated, five CpG sites (X74:82%, X75:74%, X88:74%, X89:72%, and X90:74%) and one sample (F91:83%) were excluded for further analysis since the fraction of missing data was greater than 70%. Out of the 9 final sites from Beadchip for further Sequenom, only 2 sites were significantly correlated with ages of 50 females. The possible reason for the inconsistence with the Beadchip is that there was different number of samples (16 *vs.* 49). Generally, the correlation of two variables tends to decrease with the number of the sample. In a special case that has only two samples, the absolute value of pearson coefficient is always 1. Therefore, it is reasonable that the correlation of two variables in small sample experiment (i.e. in Beadchip with 16 samples) is larger than that in large sample experiment (i.e. in Sequenom with 49 samples). To obtain age-associated sites from the large number of sample in a wide age range, we used Pearson correlation coefficients R > 0.5 or R < −0.5 as the criteria and finally obtained 11 CpG sites with the largest p-values being 0.00013 (Fig. 2b) These sites were associated with four genes including *ADAR*, *AQP11*, *ITGA2B*, and *PDE4C* (Table 1, Supplementary Table 7), among which methylation of *ITGARB* and *PDEC* have been identified to be the age-associated marker by 10 other similar reports^14^,^16^,^17^,^18^,^19^,^20^,^21^,^22^,^23^,^24^. Moreover, our studies demonstrated for the first time that CpG methylated sites residing in *ADAR* and *AQP11* genes were significantly associated with age. Specifically, among 6 CpG methylated sites related to these two genes, five sites were novel and only one site (X21) was assigned to a formal Illumina ID (cg03920003; Supplementary Table 7). Additionally, four CpG sites residing in *ITGA2B* and *PDE4C* without assignment of Illumina ID were also detected to be associated with age in our studies.

### Age prediction using different models

**Figure 3:**
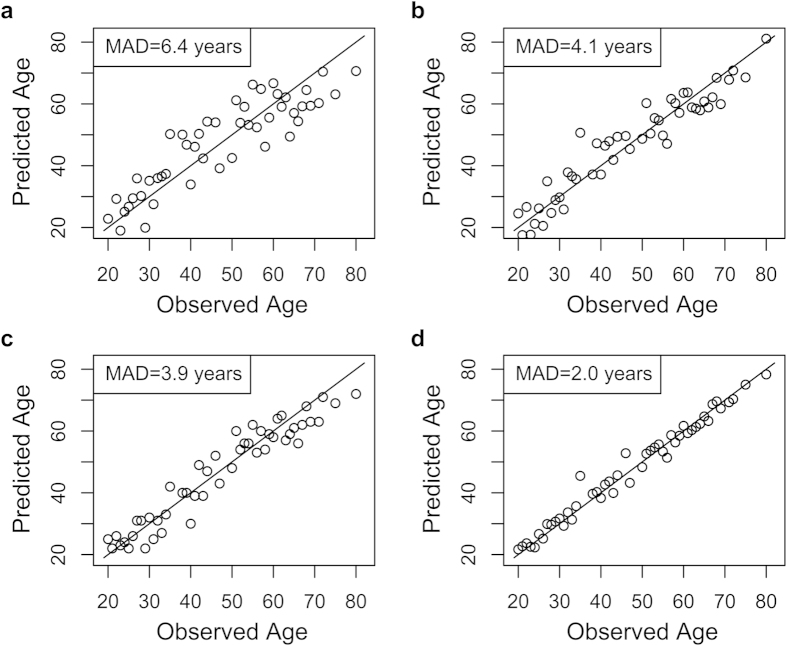
Age prediction using four models. (**a**) Multivariate linear regression model. (**b**) Multivariate nonlinear regression model. (**c**) Back propagation neural network model. **(d)** Support vector regression model. Using 11 CpG sites selected from Sequenom MassARRAY results in 49 females, the mean absolute deviation for each method was 6.4, 4.1, 3.9, and 2 years, respectively.

To build a robust predictor, we used four different models, viz., multivariate linear regression, multivariate nonlinear regression, back propagation (BP) neural network, and SVR, to test the accuracy of 11 CpG sites selected from the Sequenom dataset in age prediction (Fig. 3). We first analyzed the power of 11 CpG sites using multivariate linear regression models, and found out the age estimation error (mean absolute deviation; MAD) was 6.4 years (Fig. 3a). To further decrease the MAD, a non-linear model that might be more robust to describe the relationship between DNA methylation and age was chosen for further studies. The results showed that using the same CpG sites as in multivariate linear regression analysis, the MADs were reduced to 4.1 years for multivariate nonlinear regression (Fig. 3b), 3.9 years for BP neural network (Fig. 3c), and 2 years for SVR (Fig. 3d), respectively. Specifically for the SVR model, both the actual and predicted ages approach the diagonal (Y = X) gradually and are distributed tightly along the diagonal, indicating that SVR is the more appropriate model for age prediction (Fig. 3).

**Figure 4:**
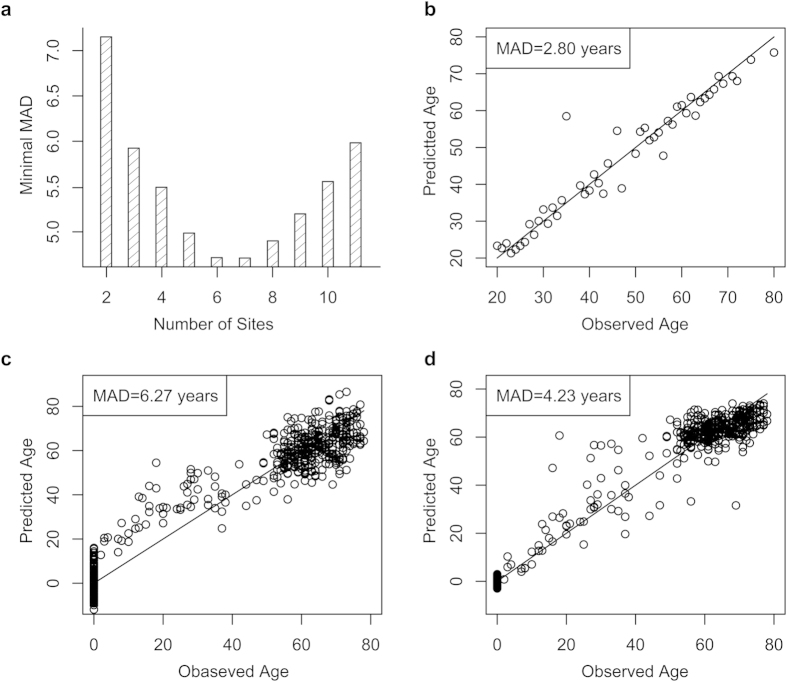
SVR is superior to linear regression in age prediction. (**a**) The minimal MAD of predicted age as a function of the number of sites that compose the independent variables. The 11 CpG sites selected from the Sequenom MassARRAY dataset were combined to one to 11 independent variables. SVR model fit on all but one sample, and the minimal MAD of the predicted age was observed for a given number of independent variables. (**b**) Predicted versus observed age of all 49 subjects, using SVR model by six markers. MAD of 2.8 years was observed, which is slightly higher than that obtained by 11 markers. (**c**) Predicted versus observed age using multivariate linear regression by three DNA methylation markers obtained from a recent study^18^. The original BeadChip data of these three sites were extracted to predict age by using a multivariate linear regression model, and an MAD of 6.27 years was obtained. (**d**) Predicted versus observed age using SVR by three DNA methylation markers obtained from a recent study^18^. MAD of 4.23 years was obtained, which is better than the MAD obtained when using a multivariate linear regression (panel C), and better than the MAD obtained when using a multivariate linear regression based on pyrosequencing data in the published study (5.4 years)^18^.

To further validate the accuracy of estimated age, we performed a leave-one-out analysis, where SVR was fit on 48 blood samples and the predicted ages were correlated with the observed ones from the left-out sample. Although MAD was as low as 2 years based on the 11 methylated CpG sites in 49 females using the SVR model, MAD was increased to about 6 years when validated with the leave-one-out model (Fig. 4a). Therefore, the SVR model using 11 methylation markers has an over-fitting problem. To test the possibility of using even fewer number of CpG sites without loss of prediction accuracy, we tried all the combinations of 11 CpG sites to predict age by using the leave-one-out method. For a given number of CpG sites (i.e. 2), the minimal MAD in age prediction was determined based on their combinations and the CpG sites with the minimal MAD were selected (Fig. 4a). Interestingly, the minimal MADs of six and seven methylation sites was 4.72 and 4.71 years, respectively, which were much lower than that obtained by using 11 markers. Considering the balance in prediction accuracy and number of CpG sites, six methylated sites with MAD of 4.72 years including X25, X28, X77, X92, X93, and X95 were selected as the more suitable set used for identifying a suspect or victim in an actual criminal investigation, even though the MAD value based on these six markers in 49 samples by SVR was 2.8 years which is slightly higher than that obtained from 11 CpG sites (Fig. 4b). Using the 6 sites as the final prediction markers, the accuracy of the SVR model was validated with an independent cohort of 10 females. The MAD of the blind test was 5.1 years (Supplementary Table 8).

Our studies demonstrated that SVR is suitable to other models in predicting age by using DNA methylation as marker. Because age can be tracked to less than 5 years with just three pyrosequencing methylated CpG sites from the blood DNA samples by using a multivariate linear regression model^18^, we speculated that the SVR model could further narrow the difference between actual and predicted ages with these three sites. However, we could only access the original BeadChip data of 102 CpG sites without any sexual information, from which we extracted the methylation profiles of the three CpG sites. We observed that the MAD value was 6.27 years when using multivariate linear regression to predict age (Fig. 4c), whereas it was reduced to 4.23 years when choosing SVR as the analyzing model (Fig. 4d) which is less than that from a multivariate linear regression analysis on both the BeadChip data (Fig. 4c) and the pyrosequencing data in the original study (5.4 years)^18^. In addition, we used leave-one-out method to compare the validation efficiency of multivariate linear regression model and SVR model based on these 102 markers. The MAD of leave-one-out of multivariate linear regression model is 10.9 years while that of SVR model is 8.1 years, further supporting SVR as a superior model in DNA methylation-based age prediction.

## Discussion

Accurate age estimation is a difficult but necessary step during forensic investigations, and various methodologies have been reported to tackle this challenging issue. The traditional method of age estimation through morphology of the skeleton and teeth in forensic science is limited due to the often corrupted or dismembered pieces of victims in criminal cases. Additionally, age prediction using several molecular biomarkers that have been shown to be correlated with age, such as telomere length, mitochondrial DNA mutation, and amino acid racemization, are often perplexed by the fact that their levels are largely influenced by the biological regulatory process in each individual, and consequently, the deviations in age estimation based on these molecular methods are often too high to be suitable for forensic practice.

Epigenetic modification such as DNA methylation has recently been shown to correlate with age^12^. Since then, a number of studies have chosen DNA methylation as the marker in age prediction and demonstrated a reduced estimation error^16^,^17^,^18^. Thus, DNA methylation becomes a convenient and more accurate marker for age estimation in forensic science. However, DNA methylation is highly divergent between populations, which may be due in large part to a combination of differences in allele frequencies, complex epistasis or gene X environment interactions^25^. Other external factors such as physical activity, diet, and sun exposure have been proposed to have a long-term influence on epigenetic modifications^26^,^27^. Moreover, most of the previous reports are focused on Caucasians^17^,^19^, the findings from which are probably inapplicable to other populations mainly due to the existing difference in population-specific DNA methylation profiles^25^. Although eight DNA fragments identified from 40 blood samples have been shown to correlate with age in Chinese^28^, whether or not common or Chinese population-specific DNA methylation sites that can be used for age prediction exist is largely unknown.

DNA methylation is influenced by gender^29^ and environmental exposures such as smoking and drinking^30^. Both sex-specific CpG sites on sex chromosomes and differentially methylated CpGs between males and females on autosomes have been demonstrated^31^,^32^. As such, gender differences must be considered when selecting age-specific methylation sites. However, females, rather than males, were more likely to fall prey to criminal activities or be dismembered by criminals, and half of the victims are young women in their 20s to 30s. Among the 24 dismembered homicide cases analyzed in Institute of Forensic Science, Shanghai Public Security Bureau from 2005 to 2012, victims were female in 16 cases with an average age of 34^33^. Another recently study reported that 78.7% of female victims were first raped before age of 25 years, investigated from 23 million women and 2.0 million men that have been raped during their lifetime^34^. Therefore, our original sampling in this study focused on females 20 to 32 years of age. Moreover, the sampling from monozygotic twin pairs in this study will largely minimize the influence from environment since monozygotic twins usually have the similar growing environment, including diet and education. By carrying out Sequenom in 50 extra females with ages of 20 to 80s, we further identified several novel age-associated CpG sites that were residing in *ADAR*, *AQP11*, *ITGA2B*, and *PDE4C*. *ADAR*, a RNA editor, encodes the enzyme that destabilizes double-stranded RNA through conversion of adenosine to inosine. Despite no studies have systemically addressed the role of *ADAR* in the aging process, mutations in two RNA editing genes *ADARB1* and *ADARB2* were found to be associated with extreme old age in human and with lifespan in *C. elegans*, suggesting that RNA editors maybe an important regulator of aging^35^. As a potential prognostic biomarker in human glioma whose incidence increases with age^36^, PDE4C exhibited promoter hypermethylation in high-grade glioma samples and hypomethylation in low-grade glioma samples with the reverse expression levels^37^, which suggests the potential correlation between PDE4C and age. Nevertheless, the possible correlation of age with the other two genes, *APQ11* and *ITGA2B*, remains to be investigated.

To establish a rapid and reliable age prediction model in forensic practice, it is ideal to use a less number of CpG sites while keeping the MAD value at least. In many bioinformatics studies, linear regression is used to build regression models owing to its speed, interpretability, and simplicity of use^16^,^19^. However, the correlation between DNA methylation and age is too complicated to be considered as linear, which may not be simply explained in linear regression. And the high-dimensional methylation data is another challenge for prediction. The simple linear regression is to fit a linear function to make the sum of absolute deviations of each point to the line minimum (Supplementary Fig. 2a). The points on the line do not contribute the linear function fitting, while only the points off the lines contribute to the evaluation of the fitting. As for linear regression in SVR, it generates a tolerant margin (*ε*, the margin of tolerance, the tolerant deviation) to cover points as many as possible (Supplementary Fig. 2b). A linear function is determined within the tolerant margin with the least possible slope^38^. However, not all points are covered in the tolerant margin. In this case, the tolerant margin can be adjusted and the deviation to the margin (ξ) for each point outside the margin is punished (Supplementary Fig. 2b). Therefore, only points outside the margin contribute to the evaluation of the fitting. And the final linear function of SVR makes the sum of slope and the deviation to margin minimum. For nonlinear regression in SVR, data is transformed to linear regression on a high dimensional feature space using kernel function^39^ (Supplementary Fig. 2c). In addition, SVR has multiple kernel functions that can deal with various types of data.

There are three main advantages of SVR predicting age. Firstly, because of the various kernel functions, SVR can adopt the complex correlation between DNA methylation and age to promote the effect of prediction and provides an exact regression function that neural network cannot provide. Another advantage of SVR is that finding the maximum margin hyperplanes makes SVR has better accuracy both on the training data and the future data^40^, which cannot be performed by simple linear regression. Moreover, as the insensitivity of dimensions, SVR is also an effective predictor to deal with the high-dimensional methylation data^40^,^41^ and greatly improves the calculation speed than multivariate regression with ergodic processes. In this study, we have compared four different regression methods and found that SVR is the best-fit prediction model for age. Therefore, the core of SVR, the kernel function, has the ability of process big data and flexibility in modeling data of varied nature, including two objects in a quantitative measure of similarity as well as data not having a clear vectorial representation (for example, DNA/RNA, sequences of proteins, protein structures) by constructing nonlinear decision rules on the basis of linear algorithms^42^.

In summary, we have identified 11 CpG sites that are highly associated with age in Chinese Han population, six out of which has successfully used for SVR platform-based age prediction with a much improved prediction accuracy and a substantially reduced MAD value. Considering the specific nature of forensic samples that are found at crime scenes, the feasibility of DNA methylation detection in more forensic samples, such as bloodstain, tissue and degraded DNA as well as the application of this novel model in these forensic samples should be further evaluated.

## Methods

### Ethics statement

All experiments of this study were carried out in accordance with the approved guidelines of Review Board, Institute of Forensic Sciences, Ministry of Public Security of China with approval number 2014FG03.

### Sample collection

Blood samples were collected from eight pairs of female monozygotic twins (aged between 21 and 32 years) and 50 non-related healthy female volunteers (aged between 20 and 80 years) with EDTA-Vacutainer tubes (Tiangen Biochemical Technology Inc). All subjects were Han Chinese without acute or chronic medical illnesses. We obtained participants’ informed consent for all samples collected. Ethical approval was received from the Review Board, Institute of Forensic Sciences, Ministry of Public Security of China. The blood samples were stored at −20 °C until processed.

### DNA extraction and bisulfite conversion

Genomic DNA was extracted from blood samples by QIAamp DNA Blood Midi kit (QIAGEN), according to the manufacturer’s protocol. DNA quantification was performed using NanoDrop® ND-2000 spectrophotometer (Thermo Scientific). According to the methylation protocol guide, 1 μg of genomic DNA was bisulfite converted using the EZ-methylation kit (Zymo Research), following Illumina (Illumina) or Sequenom (Sequenom) platform-recommended incubation conditions. Each sample was eluted with 40 μl nuclease-free water.

### Illumina Infinium HumanMethylation450 BeadChip microarray analysis

Bisulfite-converted DNA samples from eight pairs of female monozygotic twins were transferred to a 96-well microplate, and whole genomes were amplified. After being fragmented and resuspended, DNA samples were hybridized to Illumina HumanMethylation450 BeadChip (Illumina). DNA was extended by adding labeled nucleotides. BeadChips were scanned by iScan reader (Illumina), and beta values were detected using GenomeStudio software. The beta value indicates the DNA methylation level, which ranges from 0 to 1. The raw data has been deposited in NCBI’s GEO under accession number GSE65638.

### Association Testing

Methylation fraction values (beta value) having a detection p value greater than 0.01 were set to “missing”. The missing values were imputed with the KNN approach (ten nearest markers) using R “impute” package^43^. Association tests for trends in methylation status with age were performed with the linear regression model, and the Student’s *t*-test statistic for all CpG sites after the missing values were imputed. CpG sites with FDR < 0.01 were identified as sites that were significantly correlated with age and were used for Sequenom detection.

### Functional Analyses

The functional classifications of genes that were correlated with the age-associated CpG markers were assigned according to Gene Ontology (GO) using DAVID online software^44^ against genes corresponding with the rest of total loci. Functional classifications with p < 0.05 were identified as the enriched functions.

### DNA methylation level detection using Sequenom MassARRAY platform

Detection of DNA methylation levels of specific sites was performed by Sequenom MassARRAY platform (Sequenom). Primers were designed by EpiDesigner online tool (http://www.epidesigner.com) for target sequences. T7 promoter-tag (5′-cagtaatacgactcactatagggagaaggct-3′) was added to the reverse primer, and a 10mer tag (5′-aggaagagag-3′) was tagged to the forward primer to balance TM. Bisulfite-converted DNA samples of 50 non-related healthy female volunteers were amplified in a 384-well microplate. PCR products were cleaned by Shrimp Alkaline Phosphatase (SAP) treatment. As the single-stranded copies of double-stranded DNA, RNA molecules were transcribed by T7 promoter-tag for mass spectrometric detection. After *in vitro* transcription and thymine-specific cleavage, RNA molecules were digested into small pieces. Methylated and un-methylated template DNA was easily discriminated based on the mass difference of each fragmented RNA. The DNA methylation level was calculated by comparing the mass signal intensity. The methylation level analysis was undertaken by EpiTYPER® software Version 1.2.

### Aging Model

Pearson correlation coefficients (R) between ages and methylation levels were calculated for selected sites detected by Sequenom MassARRAY to identify the age-associated CpG sites (R < −0.5 or R > 0.5) that were further used to build the prediction model. Multivariate linear regression, multivariate nonlinear regression, BP neural network, and support vector regression models were performed to build the best age prediction model. The multivariate linear regression was fit in R using the ‘lm’ command with default settings. For multivariate nonlinear regression model, the quadratic multiple regression model was implemented in Matlab software using the ‘nlinfit’ function with default settings. The BP neural network was constructed using Clementine software (ver. 12.0) with the quick method. The training of the BP neural network stopped on 1000 cycles. R package ‘e1071’^45^ was implemented to build SVR model with the parameters “Cost = 2, gamma = 0.1 and epsilon = 0.1”. Leave-one-out model was used to evaluate the accuracy of the SVR model.

## Additional Information

**How to cite this article**: Xu, C. *et al.* A novel strategy for forensic age prediction by DNA methylation and support vector regression model. *Sci. Rep.* **5**, 17788; doi: 10.1038/srep17788 (2015).

## Supplementary Material

Supplementary files

Supplementary Dataset 1

Supplementary Dataset 2

Supplementary Dataset 3

## Figures and Tables

**Table 1:** Genes correlated with the 11 age-associated CpG sites obtained from the BeadChip dataset and their concordance in published studies.

Gene	Sites	Concordance in reference 14,16, 17, 18, 19, 20, 21, 22, 23, 24^*^
ADAR	X21, X25, X27, X28	/
AQP11	X68, X70	/
ITGA2B	X76, X77	ref. 14,17,18,23,24
PDE4C	X92, X93, X95	ref. 14,16, 17, 18,22,24

^*^indicates that only genes correlated with age-associated CpG sites were identified by the published studies.
