# Supplementary material for: A novel strategy for forensic age prediction by DNA methylation and support vector regression model
Source: Sci Rep. 2015 Dec 4;5:17788. doi: 10.1038/srep17788 (PMC4669521; doi:10.1038/srep17788)
Supplement: Supplementary files [file srep17788-s1.pdf]

A novel strategy for forensic age prediction by DNA methylation and support vector regression model

Chengxu[1,3,*], Hongzhu Qu[2,*], Guangyu Wang[2,*], Bingbing Xie[2], Yi Shi[1], Yaran Yang[2], Zhao Zhao[1], Lan Hu[1], Xiangdong Fang[2], Jiangwei Yan[2, §] & Lei Feng[1, §]

[1]Institute of Forensic Science, Key Laboratory of Forensic Genetics, Ministry of Public Security, Beijing, China

[2]Key Laboratory of Genome Sciences and Information, Beijing Institute of Genomics, Chinese Academy of Sciences, Beijing, China

[3]People's Public Security University of China, Beijing, China

[*]These authors contributed equally to this work.

[§]Corresponding authors:

J.Y. (yanjw@big.ac.cn)

L.F. (fengleink@163.com)

Supplementary Table 1 Age of samples

Female_ID	Twins?	Age	Experiment
D15, D16	Y	21	Illumina HumanMethylation450 BeadChip
D13, D14	Y	22	Illumina HumanMethylation450 BeadChip
D25, D26	Y	22	Illumina HumanMethylation450 BeadChip
D21, D22	Y	25	Illumina HumanMethylation450 BeadChip
D3, D4	Y	26	Illumina HumanMethylation450 BeadChip
D5, D6	Y	30	Illumina HumanMethylation450 BeadChip
D9, D10	Y	22	Illumina HumanMethylation450 BeadChip
D19, D20	Y	32	Illumina HumanMethylation450 BeadChip
F78	N	20	Sequenom Massarray
F79	N	21	Sequenom Massarray
F80	N	22	Sequenom Massarray
F81	N	23	Sequenom Massarray
F82	N	24	Sequenom Massarray
F83	N	25	Sequenom Massarray
F87	N	26	Sequenom Massarray
F89	N	27	Sequenom Massarray
F84	N	28	Sequenom Massarray
F85	N	29	Sequenom Massarray
F86	N	30	Sequenom Massarray
F88	N	31	Sequenom Massarray
F90	N	32	Sequenom Massarray
F91	N	32	Sequenom Massarray
F92	N	33	Sequenom Massarray
F13	N	34	Sequenom Massarray
F14	N	35	Sequenom Massarray
F15	N	38	Sequenom Massarray
F17	N	39	Sequenom Massarray

Supplementary Table 1 Age of samples (continued)

Female_ID	Twins?	Age	Experiment
F18	N	40	Sequenom Massarray
F20	N	41	Sequenom Massarray
F21	N	42	Sequenom Massarray
F22	N	43	Sequenom Massarray
F23	N	44	Sequenom Massarray
F24	N	46	Sequenom Massarray
F25	N	47	Sequenom Massarray
F27	N	50	Sequenom Massarray
F28	N	51	Sequenom Massarray
F29	N	52	Sequenom Massarray
F30	N	53	Sequenom Massarray
F31	N	54	Sequenom Massarray
F33	N	55	Sequenom Massarray
F34	N	56	Sequenom Massarray
F93	N	57	Sequenom Massarray
F94	N	58	Sequenom Massarray
F95	N	59	Sequenom Massarray
F96	N	60	Sequenom Massarray
F97	N	61	Sequenom Massarray
F98	N	62	Sequenom Massarray
F99	N	63	Sequenom Massarray
F100	N	64	Sequenom Massarray
F101	N	65	Sequenom Massarray
F102	N	66	Sequenom Massarray
F103	N	67	Sequenom Massarray
F104	N	68	Sequenom Massarray
F105	N	69	Sequenom Massarray
F106	N	71	Sequenom Massarray
F107	N	72	Sequenom Massarray
F49	N	75	Sequenom Massarray
F113	N	80	Sequenom Massarray

Supplementary Table 3 Functional Enrichment of genes corresponding with 2,965 age-associated CpG sites

GO_ID	Annotation	P-value
GO:0005509	calcium ion binding	0.00632
GO:0016278	lysine N-methyltransferase activity	0.00637
GO:0016279	protein-lysine N-methyltransferase activity	0.00637
GO:0018024	histone-lysine N-methyltransferase activity	0.00637
GO:0046872	metal ion binding	0.00705
GO:0043169	cation binding	0.01032
GO:0042054	histone methyltransferase activity	0.01174
GO:0043167	ion binding	0.01784
GO:0050681	androgen receptor binding	0.01971
GO:0008276	protein methyltransferase activity	0.02189
GO:0008170	N-methyltransferase activity	0.02598
GO:0035257	nuclear hormone receptor binding	0.02712
GO:0035258	steroid hormone receptor binding	0.03020
GO:0005083	small GTPase regulator activity	0.03028
GO:0022843	voltage-gated cation channel activity	0.03107
GO:0030145	manganese ion binding	0.03217
GO:0030528	transcription regulator activity	0.03956
GO:0022836	gated channel activity	0.04519
GO:0004653	polypeptide N-acetylgalactosaminyltransferase activity	0.04682
GO:0022832	voltage-gated channel activity	0.04968
GO:0005244	voltage-gated ion channel activity	0.04968

Supplementary Table 5 Primer information of 11 CpG sites for sequenome Massarray

TargetID	Primer	Forward primer	Reverse primer	CpG sites #	Target length (bp)
cg09824328	P1	aggaagagagTTTAGGTGAGTTTATGGTGATTTGT	cagtaatacgactcactatagggagaaggctATTCAAAACCAACCTAACCAAAATA	10	402
cg07735952	P2	aggaagagagTGAAGGTGGAGATGGTATAGTAGGT	cagtaatacgactcactatagggagaaggctCAACTAATCAAACCAATTCCCTAAA	10	483
cg17218026	P3	aggaagagagATTTTTTTGTAGGTTGGGTTTTGTT	cagtaatacgactcactatagggagaaggctAACATCCATCTTCCCTAACAAACTA	8	373
cg22801896	P4	aggaagagagTGAGATTTTTGTTTTTAGAGGATATGG	cagtaatacgactcactatagggagaaggctAATAAAACAAACCAACAAACCAAAA	10	220
cg25923729	P5	aggaagagagTGTTTTTTGATTTTTTATGAGTAGGAGA	cagtaatacgactcactatagggagaaggctAAACTTAACTTCCAAAATTTCCACC	1	481
cg21438057	P6	aggaagagagTTGAAGTTATTGTTAAAGTTGTTTTTGTT	cagtaatacgactcactatagggagaaggctACACCCCACAAAAAATATCCAAT	13	222
cg23982607	P7	aggaagagagGATTGGAGATTTTTAGGTGAGGAA	cagtaatacgactcactatagggagaaggctAAATTAAATCTCTCTCCCCTTCAAC	7	422
cg26975524	P8	aggaagagagGGTTAGGTTAGTAGGAGGTGGGAT	cagtaatacgactcactatagggagaaggctAAAAAAACTTCCTTAACAACCACAA	5	463
cg04518186	P9	aggaagagagTTGTGGTTTAGGTAGGGGTGTAGT	cagtaatacgactcactatagggagaaggctCCCTCACAATATTATTCAACAATCAC	8	441
cg02228185	P10	aggaagagagAAGGGAATTTTGTATTTTGTTTTTTG	cagtaatacgactcactatagggagaaggctAAACATAATCTTACCCAAAATTTTCAA	3	377
cg25809905	P11	aggaagagagTTTTGGTTTAGGTGGTAAGGAGATT	cagtaatacgactcactatagggagaaggctATATTTCCATCCAATCTTTCAACAA	3	377
cg17861230	P12	aggaagagagTTTTTTGGAGAAGTAAGGATTTTTTTT	cagtaatacgactcactatagggagaaggctTACTATCCCAAACCCCTTTCTCTAA	17	362

Supplementary Table 7 Eleven CpG sites were highly correlated with age in 50 female.

Target	r	Primer	Gene	Position	Illumina ID
X21	-0.582**	P3	ADAR	chr1:154582007	cg03920003
X25	-0.626**	P3	ADAR	chr1:154582187	/
X27	-0.521**	P3	ADAR	chr1:154582205	/
X28	-0.677**	P3	ADAR	chr1:154582288	/
X68	-0.662**	P9	AQP11	chr11:77299956	/
X70	-0.684**	P9	AQP11	chr11:77299980	/
X76	-0.565**	P11	ITGA2B	chr17:42467726	/
				chr17:42467728	cg25809905
X77	-0.590**	P11	ITGA2B	chr17:42467780	/
X92	0.624**	P12	PDE4C	chr19:18343915	/
X93	0.652**	P12	PDE4C	chr19:18343937	/
				chr19:18343941	/
				chr19:18343943	/
X95	0.593*	P12	PDE4C	chr19:18344003	/

*P<0.05, **P<0.01

Supplementary Table 8 Validation of the SVR model using 6 sites with 10 independent females.

Sample ID	Actual Age	Methylation levels of 6 sites						Predicted Age	MAD*
		X25	X28	X77	X92	X93	X95		
1	52	0.32	0.47	0.47	0.38	0.39	0.11	49.39	5.09
2	27	0.34	0.66	0.59	0.28	0.23	0.04	28.36	
3	41	0.1	0.6	0.63	0.3	0.29	0.17	51.43	
4	42	0.14	0.5	0.52	0.13	0.13	0.02	34.33	
5	31	0.23	0.51	0.66	0.17	0.15	0.19	34.18	
6	28	0.38	0.68	0.9	0.26	0.74	0.29	38.38	
7	54	0.24	0.05	0.52	0.25	0.24	0.11	48.7	
8	36	0.15	0.89	0.48	0.24	0.27	0.49	36.19	
9	42	0.27	0.64	0.6	0.3	0.26	0.04	33.75	
10	33	0.14	0.57	0.65	0.14	0.16	0.12	31.46	

***MAD = minimal absolute deviation**

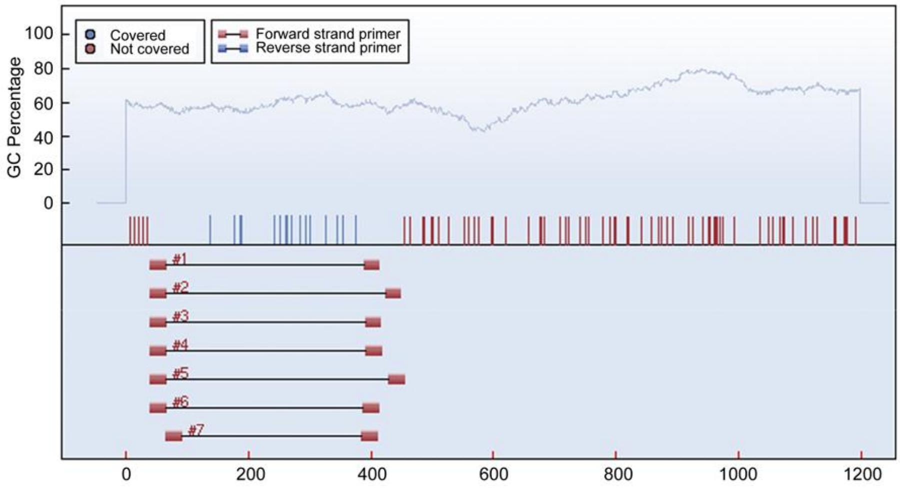

Supplementary Figure 1 The distribution of CG dinucleotides around cg11296826 (± 600bp). Vertical lines indicate CpG sites.

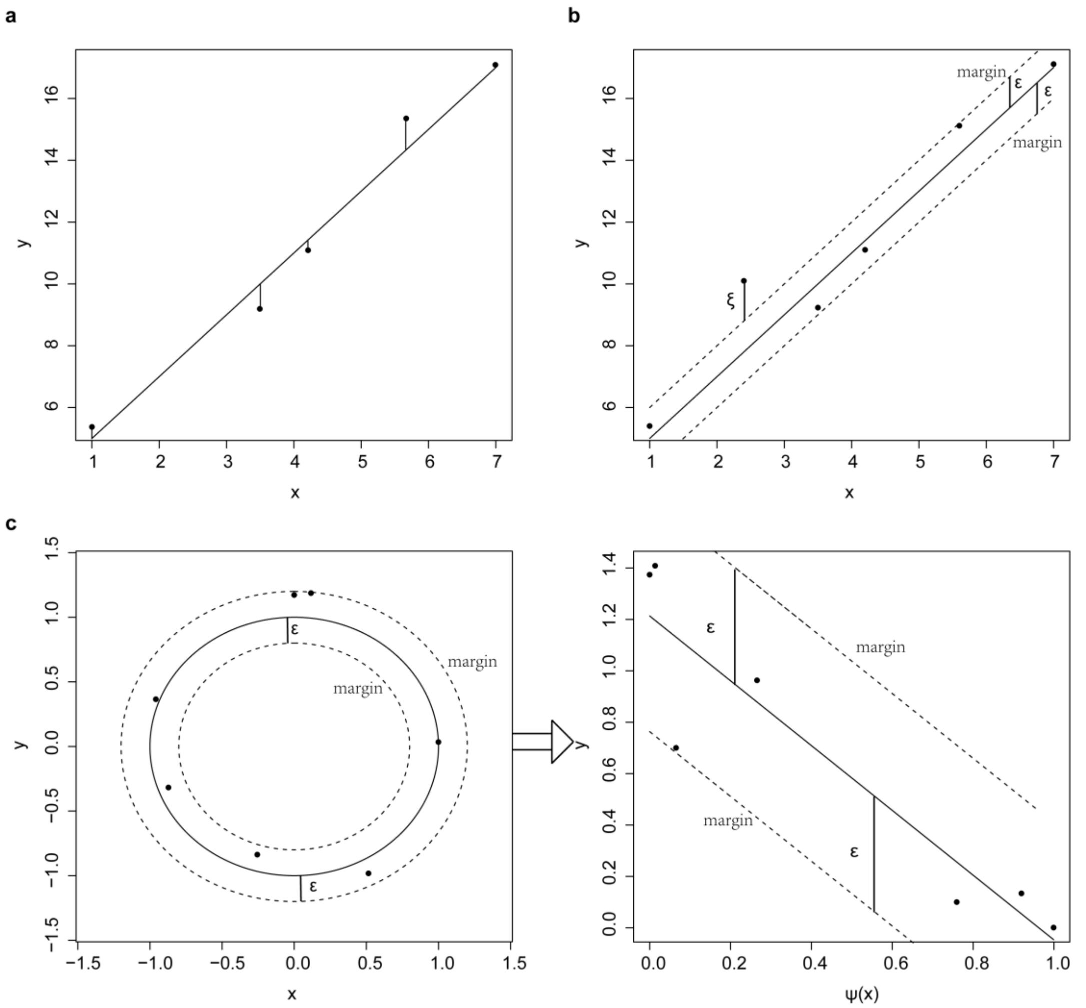

Supplementary Figure 2 The schematic of linear regression and SVR. **(a)** The schematic of linear regression. The full line is the regression function which makes the sum of absolute deviations of each point to the line minimum. Therefore, only the points off the lines contribute to the evaluation of the fitting. **(b)** The schematic of linear regression in SVR. The band between dish lines is the tolerance margin with ε accuracy. The points out of the margin are punished by ξ. The full line is the regression function that makes the sum of its slope and the deviations to margin (ξ) minimum. Points outside of the margin contributed the evaluation of the regression. **(c)** The schematic of nonlinear regression in SVR. The points (x,y) of nonlinear regression (the left figure) are transferred into a feature space (ψ(x),y) (the right figure) by a kernel function ψ(x).
